# Single-cell transcriptomic profiling reveals immune cell heterogeneity in acute myeloid leukaemia peripheral blood mononuclear cells after chemotherapy

**DOI:** 10.1007/s13402-023-00853-2

**Published:** 2023-08-24

**Authors:** Xuqiao Hu, Dongyan Cao, Zhenru Zhou, Zhaoyang Wang, Jieying Zeng, Wen-Xu Hong

**Affiliations:** 1 Shenzhen Center for Chronic Disease Control and Prevention, Shenzhen Institute of Dermatology, Shenzhen, China; 2 Second Clinical Medical College of Jinan University, First Affiliated Hospital of Southern University of Science and Technology (Shenzhen People’s Hospital), Shenzhen, China; 3 Department of Biliary-Pancreatic Surgery, the Renji Hospital Affiliated to Shanghai Jiaotong University School of Medicine, Shanghai, China; 4 State Key Laboratory of Oncogenes and Related Genes, Shanghai Cancer Institute, Shanghai Jiaotong University School of Medicine, Shanghai, China

**Keywords:** AML, scRNA-seq, PBMC, Monocyte, Chemotherapy

## Abstract

**Purpose:**

Acute myeloid leukaemia (AML) is a heterogeneous disease characterised by the rapid clonal expansion of abnormally differentiated myeloid progenitor cells residing in a complex microenvironment. However, the immune cell types, status, and genome profile of the peripheral blood mononuclear cell (PBMC) microenvironment in AML patients after chemotherapy are poorly understood. In order to explore the immune microenvironment of AML patients after chemotherapy, we conducted this study for providing insights into precision medicine and immunotherapy of AML.

**Methods:**

In this study, we used single-cell RNA sequencing (scRNA-seq) to analyse the PBMC microenvironment from five AML patients treated with different chemotherapy regimens and six healthy donors. We compared the cell compositions in AML patients and healthy donors, and performed gene set enrichment analysis (GSEA), CellPhoneDB, and copy number variation (CNV) analysis.

**Results:**

Using scRNA-seq technology, 91,772 high quality cells of 44,950 PBMCs from AML patients and 46,822 PBMCs from healthy donors were classified as 14 major cell clusters. Our study revealed the sub-cluster diversity of T cells, natural killer (NK) cells, monocytes, dendritic cells (DCs), and haematopoietic stem cell progenitors (HSC-Prog) in AML patients under chemotherapy. NK cells and monocyte-DCs showed significant changes in transcription factor expression and chromosome copy number variation (CNV). We also observed significant heterogeneity in CNV and intercellular interaction networks in HSC-Prog cells.

**Conclusion:**

Our results elucidated the PBMC single-cell landscape and provided insights into precision medicine and immunotherapy for treating AML.

**Supplementary Information:**

The online version contains supplementary material available at 10.1007/s13402-023-00853-2.

## Introduction

Acute myeloid leukaemia (AML), a highly heterogeneous clonal malignant tumor of immature myeloid haematopoietic stem cells, is characterised by increased proliferation and blocked terminal differentiation of abnormal myeloid progenitor cells in the bone marrow and other tissues [1]. In addition, AML may result from a series of genetic changes that can accumulate in haematopoietic stem cells with age or radiotherapy [2]. Currently, AML therapies mainly include chemotherapy [3, 4] and haematopoietic stem cell transplantation [5]. Although standard chemotherapy treatment has improved due to precision medicine, less than 40% of patients can achieve a 5-year survival rate, and 75% of patients die of relapse within five years of AML diagnosis, according to clinical statistics [6, 7]. Clinical AML prognosis and medication are dependent on specific gene mutations, such as *TP53, FLT3*, *NPM1*, *DNMT3A*, *IDH1*, *IDH2*, *CEBPA*, and *PHF6* or specific chromosome translocations [8, 9], which are used to grade clinical risk. The advent of immune checkpoint inhibitors has enabled the treatment of patients with tumors and has substantial benefits [10, 11]. However, the therapeutic outcomes vary greatly from individual to individual, either with drug resistance or with its intrinsic heterogeneity or dynamic immunogenic features. Therefore, an in-depth understanding of the immune landscape and microenvironment of AML patients will benefit the more accurate classification of clinical diagnosis and treatment, which eventually to achieve personalised precision.

In this study, five AML patients with a defined clinical risk grades and status were enrolled, either undergoing chemotherapy or transplantation. We used single-cell RNA sequencing (scRNA-seq) technology to analyse the transcriptomic profile of 44,950 peripheral blood mononuclear cells (PBMCs) from five AML patients and 46,822 PBMCs from six healthy donors. Using uniform manifold approximation and projection (UMAP), we identified 14 major cell clusters and characterised the cellular properties of CD4^+^ T cells, CD8^+^ T cells, natural killer (NK) cells, monocytes, and dendritic cells (DCs). In addition, we performed gene set enrichment analysis (GSEA), CellPhoneDB, and copy number variation (CNV) analysis. As a result, the expression of transcription factors involved in cell proliferation and survival was downregulated in AML patients, while the chromosomal CNV in NK and monocyte-DCs was upregulated in AML patients. Besides, we also observed a high degree of variability in the intracellular interaction network and CNV in haematopoietic stem cell progenitors (HSC-Prog) cells in AML patients. Overall, our findings clarify the single-cell immune landscape of PBMC of AML and offer insights into the development of personalised precision medicine and immunotherapy for patients with AML.

## Results

### Identification of cellular composition in AML and healthy PBMC samples

**Fig. 1:**
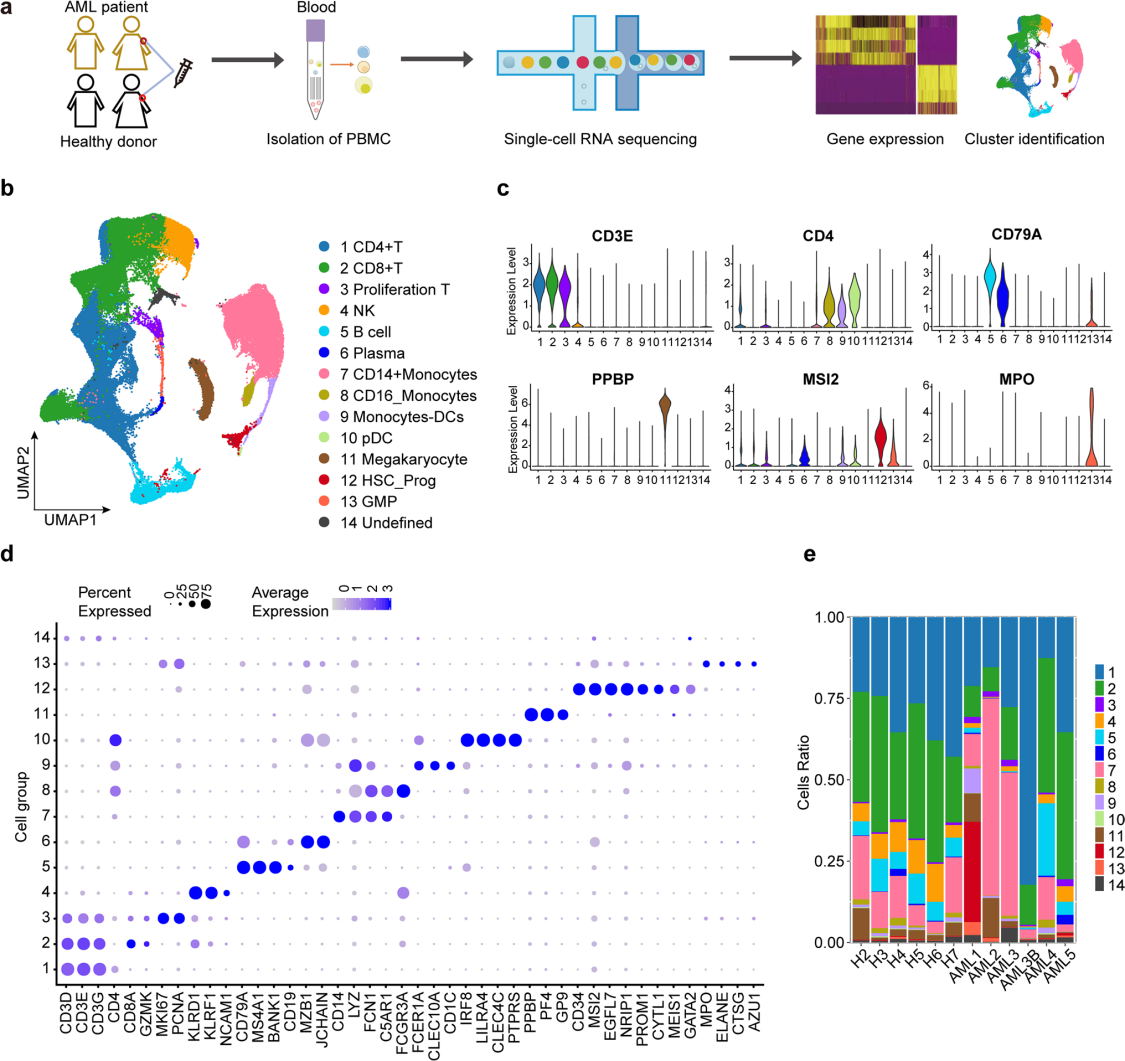
Single-cell transcriptomic analysis in AML patient PBMC cells and healthy donor PBMC cells. **a** Graphical view of the study roadmap. Single-cell suspensions were collected from five AML patients and six healthy donors, followed by scRNA-seq on the 10 × Genomics platform. **b** UMAP analysis of 91,772 qualified single cells resulted in identifying 14 major cell type clusters. Clusters are labelled in different colours and numbers. **c** Violin plots show the expression levels of some marker genes across the 14 cellular clusters. **d** Dot plots show the signature gene expression levels across the 14 cellular clusters. **e** Relative proportion of cell clusters in each AML patients or healthy donors across the main 14 cellular clusters

We performed scRNA-seq analysis of PBMC samples from five AML patients (two males and three females, 33–54 years old) and six healthy humans (three males and three females, 26–37 years old) to explore their cellular composition (Fig. 1a, Supplementary Table 1). The cohort included patients with either chemotherapy or transplantation at different stages (bone marrow suppression period or immune recovery period) and was genetically heterogeneous (Supplementary Table 1). Among then, AML3 and AML3B derived from the same patient, but they are in different stages after chemotherapy. After an initial quality control assessment and doublet removal using the Seurat R package and DoubletFinder, we obtained a total of 91,772 cells, with 44,950 cells from healthy donors and 46,822 cells from AML patients, for single-cell transcriptome analysis. The final cell number ranged from 1,936 to 14,081 per sample (Supplementary Fig. 1a, Supplementary Table 2), the median unique molecular indices (UMIs) ranged from 4,001 to 10,864 per cell (Supplementary Fig. 1b), and the sequencing saturation ranged from 68.7% to 83.2% per sample (Supplementary Table 3). In addition, based on UMAP analysis, we identified 14 main clusters in parallel according to the gene profile and representative gene markers of the cell (Fig. 1b and c), *CD3D*, *CD3G*, *CD3E,* and *CD4* for CD4-T cells; *KLRF1*, *NCAM1,* and *KLRD1* for NK cells; and *LYZ*, *CD14*, *FCN1,* and *C5AR1* for CD14^+^ monocyte cells.

The relative proportions of cell clusters and the relative expression levels of cluster-specific markers in each cluster were also confirmed (Fig. 1d, e and Supplementary Fig. 2 and 3). There was high heterogeneity in the proportion of cell clusters among AML samples compared to healthy samples that showed consistent immune cell compositions (Fig. 1e, Supplementary Fig. 4, and Supplementary Table 4). The AML1 sample who had TP53 mutant that belong to high-risk grade, had a considerably higher number of HSC-prog cells, which were rarely detected in the other AML samples. The excessive number of HSC-prog cells may be related to AML relapse, as previous studies have reported that rare leukaemia cells with stem cell characteristics such as self-renewal ability and drug resistance may contribute to the maintenance and relapse of the disease [12, 13]. Compared to healthy donors, AML2 and AML3 had higher proportions of CD14^+^ monocytes, suggesting their role in inhibiting the activation of T cells and promoting AML blasts survival [6]. Compared to AML3B, the proportion of CD14^+^ monocytes in AML3B decreased obviously. In addition, AML3B, showed an 82% increase in CD4^+^ T cells compared to healthy donors, indicating an immunostimulatory effect of chemotherapy in promoting the formation of CD4-T cells [14]. After hemiallogeneic hematopoietic stem cell transplantation, AML4 had a similar cell type composition with healthy donors, which proved stem cell transplantation was an effective method to cure AML4. Similar to AML3B, AML5 also had major T cell, which might attribute to their myelosuppression status. Collectively, the results of our study reveal heterogeneity in immune cell types and proportions in AML patients and suggest potential relationships with their clinical status under chemotherapy or transplantation that are compatible with existing AML progression and maintenance theories.

### Nine CD4^+^ T and CD8^+^ T subtypes shaped the immune landscape in AML

**Fig. 2:**
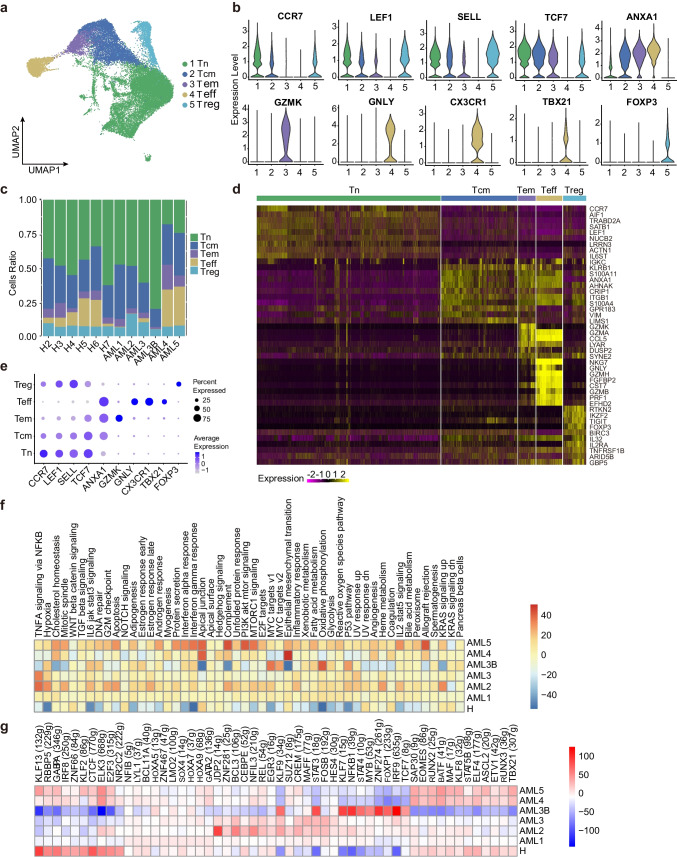
Immune landscape of CD4^+^ T cells in AML and healthy samples. **a** Five main CD4^+^ T cell subclusters were identified via UMAP analysis. **b** Violin plots show the expression levels of marker genes across the five subclusters of CD4^+^ T. **c** Proportion of five CD4^+^ T subsets in total CD4^+^ T cells in each AML patient or healthy donor. **d** Heatmap of gene expression and cellular cluster distribution among the five CD4^+^ T subclusters. **e** Dot plots showing the 10 signature gene expression levels across the five cellular clusters. **f** Heatmap of the 50 hallmark gene sets in the MSigDB database among AML patients and healthy donors of Treg cell. **g** Heatmap of AUCell tvalue transcription factor (TF) in CD4^+^ T cells among AML patients and healthy donors. Red and blue represent upregulated and downregulated TFs, respectively

To further characterise the intrinsic structure and potential functional subtypes of the overall T cell populations, we performed spectral clustering on all T cells using UMAP [15]. As a result, we identified nine stable subclusters, including five subclusters of CD4^+^ cells and four subclusters of CD8^+^ cells, each expressing their unique signature genes. The signature gene expression patterns in different subclusters of CD4^+^ and CD8^+^ T cells are shown in Supplementary Fig. 5a and Supplementary Fig. 6a. The relative cluster-specific marker expression levels in the five cellular subclusters of CD4^+^ T have been presented in Fig. 2b, d and e. For example, cells that specifically expressed naive marker genes such as *LEF1*, *SELL,* and *CCR7* represented naïve T cells (Tn) [16]. In contrast, the central memory T cell (Tcm) cluster was represented by cells expressing *TCF7*, *ANXA1, LEF1,* and *SELL* genes [17] and is commonly associated with central memory and tumor inhibition function [18, 19]. Other clusters include the effector memory cell (Tem) cluster that highly expressed *GZMK* [20]; the effector T cell (Teff) cluster that specifically expressed ‘effector’ genes such as *ANXA1*, *GNLY*, *CX3CR1*, and *TBX21* [21]; and the regulatory T cell (Treg) cluster that expressed *LEF1*, *SELL,* and regulatory marker *FOXP3*. The proportion of cells expressing cluster-specific markers was shown in Fig. 2c.

We observed high variation in the number of CD4^+^ Tem, Teff, and Treg in AML patients, except for CD4^+^CCR7^+^Tn and CD4^+^TCF7^+^Tcm, which were prevalent in peripheral blood in AML and healthy samples (Fig. 2c, Supplementary Fig. 5b). The percentage of cytotoxic CD4^+^Teff varied among AML patients (Fig. 2c), sharing a low proportion in most AML patients, except in AML4 and AML5, suggesting that the enhanced functions of various cells are involved in exerting immune effects [22]. Tregs are known to contribute to the suppression of anti-leukemic activity [23], and their immunosuppressive function contributes to leukaemia progression [24]. AML3B is in myelosuppression state, we also observed a decrease in Tregs in AML3B compared to AML3, consistent with the myelosuppression effect of chemotherapy [25]. These observations suggest that CD4^+^ T cells should not be viewed as a homogeneous population in tumor samples and that the cellular constitution of CD4^+^ T cell sub-clusters are associated with AML disease conditions.

Next, we analysed the AML and healthy CD4^+^ T cells using gene set variation analysis (GSVA) and the result showed high individual heterogeneity in AML patients, significantly differing from healthy controls, as indicated in Fig. 2f. We observed downregulation of five transcription factors involved in the development of T cell immunity, including *KLF13*, *GABPA*, *RBBP5*, *ZNF66,* and *IRF8,* in most AML patients. The exception is AML3B, which upregulates transcription factors related to cell growth and differentiation, including *STAT3*, *KLF7*, *NFKB1, STAT4, MYB*, *ZNF274, FOXP1, IRF9* and *TCF7*. In adverse, these genes were downregulated in healthy donors (Fig. 2g). In addition to transcription factors, in AML samples, we identified the upregulation of genes that were reported to accelerate leukaemogenesis, including *S100A8*, *S100A9*, and *RPS26* [26, 27], and the downregulation of genes related to cancer control and defence, such as *XIST*, *GIMAP7*, *NKG7*, and *GNLY* (Supplementary Fig. 7). Among the downregulated genes, *XIST* is a non-coding RNA transcript on the X chromosome that acts as a major effector in the X-inactivation process [28]. *NKG7* is critical for controlling cancer initiation, growth, and metastasis and regulating lymphocyte granule exocytosis and downstream inflammation [29]. *GNLY* is found in cytotoxic granules of cytolytic T lymphocytes and NK cells, which are essential in defence against tumors and microbes [30]. These results reveal the modulation of CD4^+^ T cells in AML, such as suppression of CD4^+^ T cell activities via downregulation of transcription factors and genes related to cancer control and defence and upregulation of genes in CD4^+^ T cells that promote leukaemogenesis and inflammatory signalling.

### Immune landscape of CD8^+^ T

**Fig. 3:**
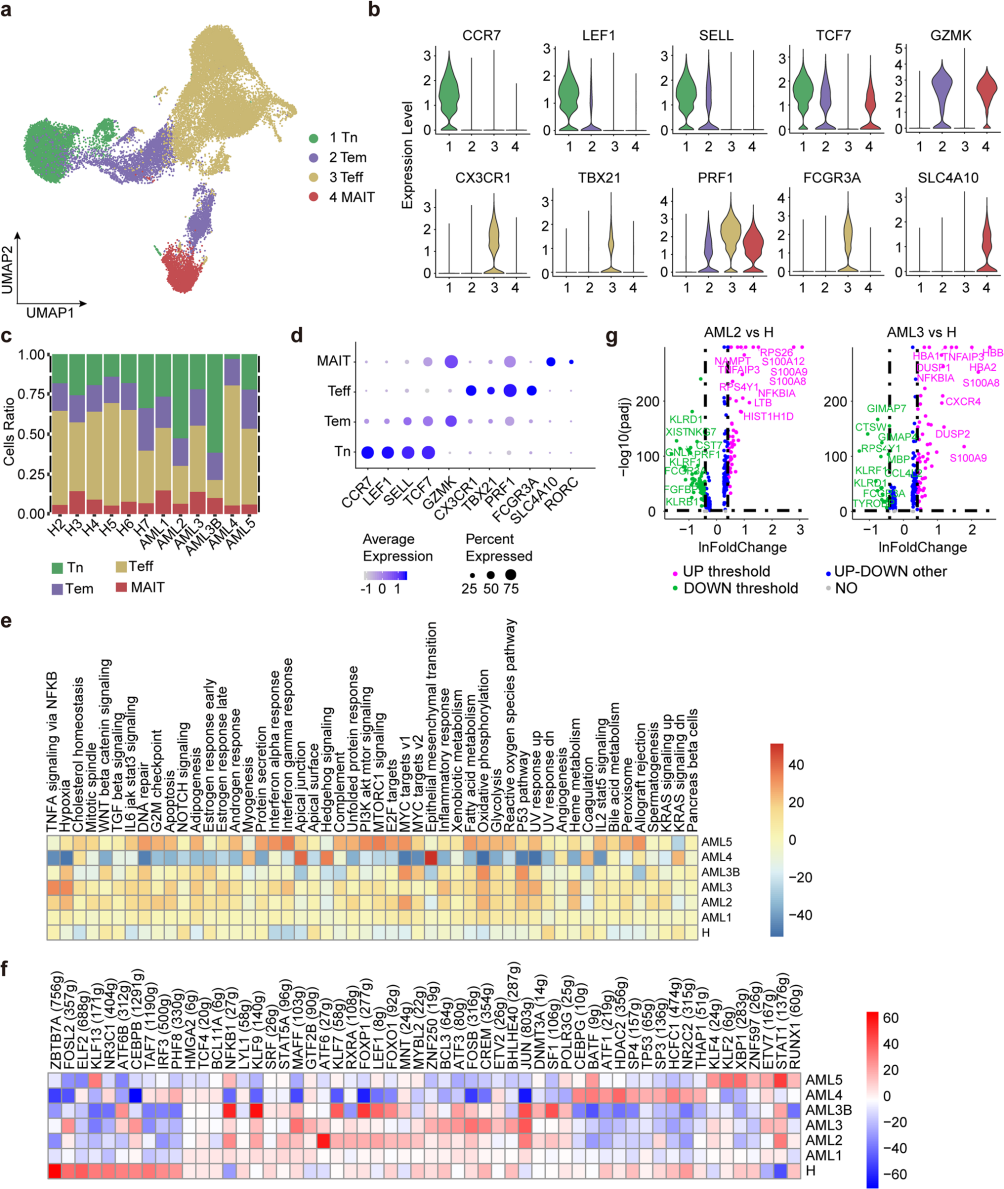
Immune landscape of CD8^+^ T cells in AML and healthy samples. **a** Four main CD8^+^ T cell subclusters were identified by UMAP analysis. **b** Violin plots show the expression levels of marker genes across the four subclusters of CD8^+^ T. **c** Relative proportion of each CD8^+^ T subcluster across all samples. **d** Dot plots show the 11 signature gene expression levels across the four cellular clusters. **e** Heatmap of the 50 hallmark gene sets in the MSigDB database among the AML and healthy donors. **f** Heatmap of AUCell tvalue of transcription factor in CD8^+^ T cells among AML patients and healthy donors. Red and blue represent upregulated and downregulated TFs, respectively. **g** Volcano plot shows differentially expressed genes in CD8^+^ T cells. Genes exhibiting significant differential expression are represented by green (downregulated) and purple (upregulated) dots, and the selected genes are highlighted

Next, we focused on marker gene expression and composition ratios of the four major CD8^+^ T cell clusters (Fig. 3a-d). CD8^+^ Teff cells highly express *CXCR3*, *TBX21*, *PRF1,* and *FCGR3A* genes, which are commonly associated with T cells with effector functions [31], while CD8^+^ Tn cells specifically express effector marker genes *CCR7*, *LEF1*, *SELL,* and *TCF7* [16], together with CD8^+^ Tem cells that express high levels of *GZMK*, similar to their CD4^+^ counterpart [20]. Notably, we observed that a CD8^+^ T cell subcluster was composed mainly of mucosal-associated invariant T cells (MAIT), characterised by the specific expression of *SLC4A10* and *RORC* (Fig. 3d). Unlike CD4^+^ T cells, we observed similar relative compositions of CD8^+^ T sub-clusters among healthy donors and patients with AML. In addition, we observed a significant downregulation of 29 transcription factors associated with immune cell homeostasis and cancer control across all AML samples, including *ZBTB7A*, *NR3C1*, *ELF2*, *KLF13*, *KLF2*, *XBP1*, *RXRA*, *FOSL2,* and *HDAC2* (Fig. 3f and 3g). Among these, *ZBTB7A* determines the fate of the haematopoietic system and regulates glycolysis, and AML patients are prone to *ZBTB7A* mutations [32]. *HDAC* is an epigenetic regulator that is tightly involved in AML aetiology and mediates the immune system regulation [33]. We then conducted GSVA analysis of CD8^+^ T subclusters to compare the differences in enriched pathways between AML patients and healthy donors. We observed an upregulation of 50 hallmark pathways related to immune reactions, cancer progression, and immunotherapy in AML patients [31], such as hypoxia, MYC target v1, oxidative phosphorylation, P53 pathway, and heme metabolism (Fig. 3e) [34, 35]. Notably, the MYC target v1 pathway is reported to be an important oncogenic pathway in cancer development [32]. We also observed variations in enriched oncogenic pathways that promote T cell growth and survival in certain AML patients. For example, AML4 demonstrated significant enrichment of genes involved in hedgehog signalling and KRAS signalling [36], while AML5 showed heightened activities of MTORC1 signalling pathways [37]. Our results demonstrate that AML promotes leukaemogenesis in CD8^+^ T cells through the upregulation of multiple hallmark pathways and highlight the need for personalised medicine to target upregulated transcription factors and oncogenic signalling pathways for AML treatment.

### Transcriptome of NK cells in AML

**Fig. 4:**
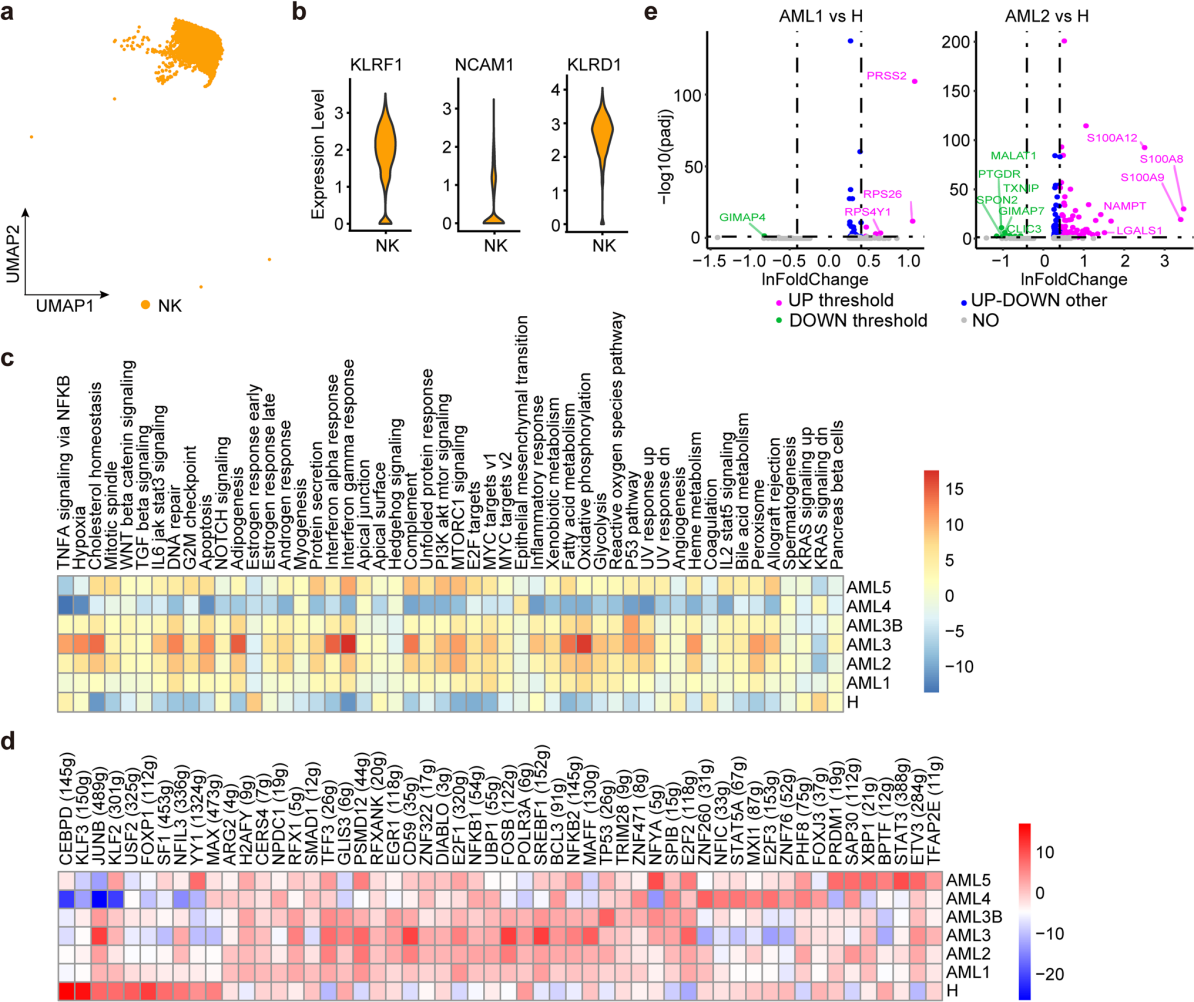
Immune landscape of NK cells in AML and healthy samples. **a** One major NK subcluster was identified by UMAP analysis. **b** Violin plots show the expression levels of three marker genes in NK cells. **c** The heatmap of 50 hallmark gene sets in the MSigDB database of NK cells. **d** Heatmap of AUCell tvalue of transcription factor in NK cells among AML patients and healthy donors. Red and blue represent upregulated and downregulated TFs, respectively. **e** Volcano plot shows genes differentially expressed in NK cells. Genes displaying significant differential expression are represented by green (downregulated) and purple (upregulated) dots, and selected genes are highlighted

AML is often accompanied by a reduction in NK cell activity in peripheral blood [38]. We obtained approximately 4,395 NK cells from all samples (Fig. 4a), marked with *KLRF1*, *NCAM1,* and *KLRD1* (Fig. 4b). Unlike T cells, UMAP spectral clustering on NK cells only results in single functional subtype showing NK cell marker gene expression homogeneity among AML patients and healthy donors. We also observed that the number of NK cells in AML patients was lower than that in healthy donors (Fig. 1e), indicating a potential role for NK cells in AML-induced suppression [38]. We then compared the DEGs in NK cells between AML and healthy donors. We observed the downregulation of 10 transcription factors in all six AML samples, including factors involved in homeostatic NK cell proliferation and survival, such as *CEBPD*, *KLF3*, *KLF2, USF2*, and *FOXP1*. (Fig. 4d). For other genes, we observed transcriptional heterogeneity among AML samples. As indicated in Fig. 4e, AML1 exhibited lower expression levels of the *GIMAP4* gene, which encodes a protein belonging to the immune-associated nucleotide subfamily and is negatively regulated by T-cell acute lymphocytic leukaemia 1 (TAL1). In contrast, AML1 exhibited higher expression levels of genes encoding ribosomal proteins, including *RPS26* and *RPS4Y1* [39], which may promote cancer metastasis and spread, and their presence is correlated with increased disease aggressiveness and poor clinical outcomes [40]. Similar to AML1, AML3B and AML4 also upregulated some ribosomal genes (Supplementary Fig. 8). In AML2 and AML3, the upregulated genes contained *S100A8* and *S100A9*, which are known to be poor prognostic indicators in AML, and elevated expression of the heterodimer *S100A8*/*S100A9* was previously reported to cause glucocorticoid resistance in MLL-rearranged infant acute lymphoid leukaemia cells [41] (Fig. 4e and Supplementary Fig. 8). AML5 displayed upregulation of several genes related to encoding immunoglobulins (*IGKC*, *IGHA1*, *ERAP2,* and *IGLC2*, Supplementary Fig. 8) that participate in indirect cytotoxicity by recognising IgG antibodies attached to target cells, which is a key mechanism of action for various clinically successful anti-tumor therapeutic monoclonal antibodies (mAbs) [42]. Based on GSVA analysis (Fig. 4c), we observed high heterogeneity in gene set enrichment among AML samples, except for AML4, which had enriched gene sets similar to those of healthy samples. AML3 and AML5 showed heightened enrichment of pathways involved in anti-tumor response and lipid metabolism, including the interferon-gamma response, oxidative phosphorylation, adipogenesis, interferon-alpha response, and fatty acid metabolism pathways. In addition, AML2 was enriched with apoptosis and MTORC1 signalling pathways, whereas AML3B was enriched with P53 signalling pathways. These findings emphasise the negative impact of AML on cell number and anti-tumor activity of NK cells, as NK cells from healthy donors are activated with higher interferon-alpha and gamma response profiles. However, our results also demonstrated the potential of AML treatment by targeting the regulation of transcription factors, ribosomal proteins, and inflammatory signalling.

### Monocyte and DC expansion in AML

**Fig. 5:**
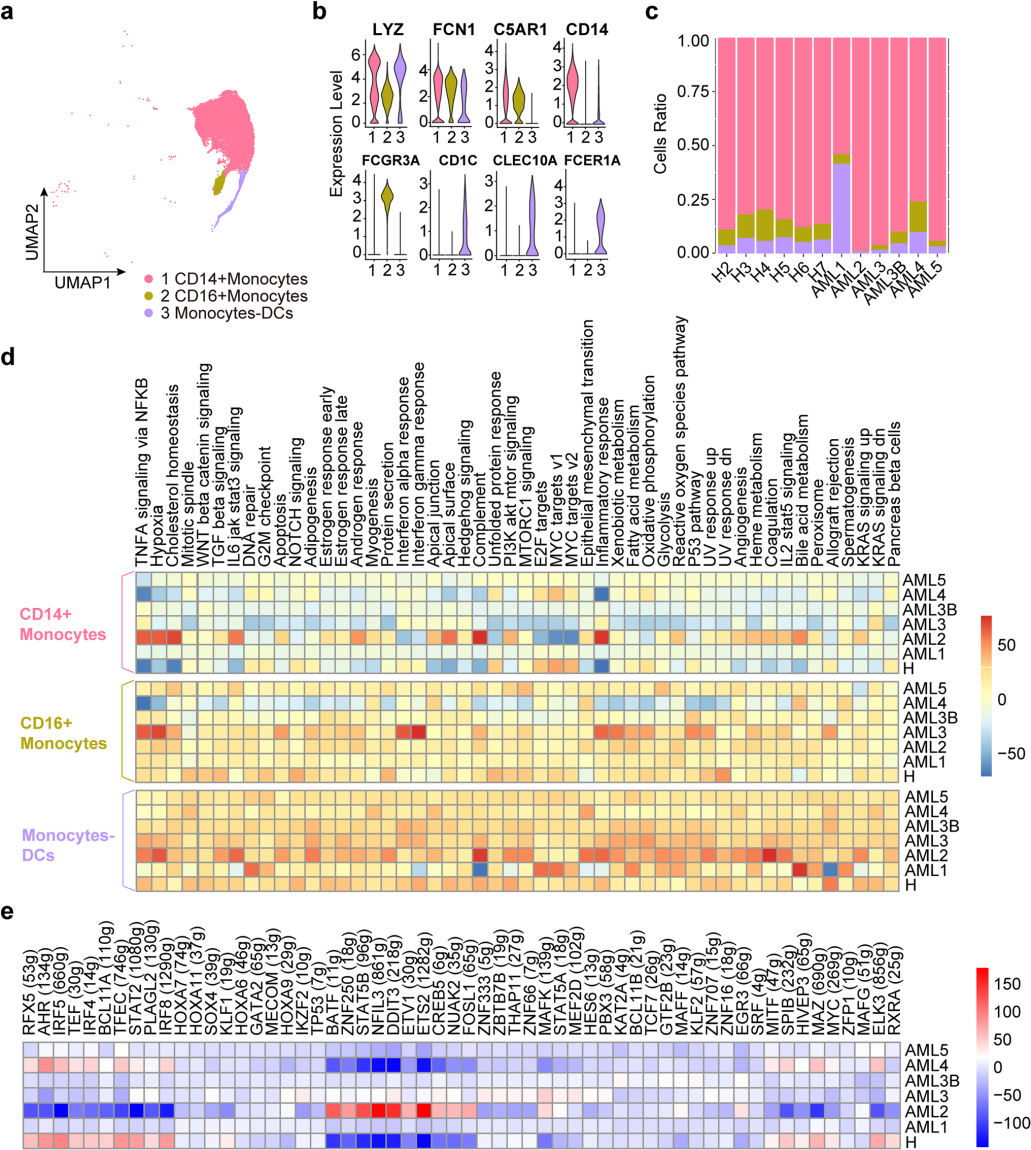
Immune landscape of Monocyte and DC cells in AML and healthy samples. **a** UMAP analysis of CD14^+^ and CD16^+^ monocytes identified in the monocytes. **b** Violin plots show the expression levels of marker genes across the three monocyte subclusters. **c** Relative proportion of CD14^+^ and CD16^+^ monocytes across the monocytes. **d** The heatmap of the 50 hallmark gene sets in the MSigDB database among the healthy and AML patients. **e** Heatmap of AUCell tvalue of transcription factor in CD14^+^ monocytes among AML patients and healthy donors. Red and blue represent upregulated and downregulated TFs, respectively

Monocytes are a heterogeneous group of cells originating from myelomonocytic precursors in the bone marrow, which contribute to host defence against pathogens and maintain normal tissue structure and function [43]. DCs, derived from monocytes, are essential for initiating and regulating immune responses. We analysed the heterogeneity of CD14^+^CD16^+^ monocytes associated with inflammation and AML disease progression in AML using UMAP analysis (Fig. 5a) [44]. We identified three major monocyte subsets in human peripheral blood based on the differences in phenotype, function, and expression of markers such as *LYZ*, *FCN1,* and *C5AR1* [45]. The subclusters included CD14^+^ monocytes expressing high levels of *CD14*, CD16^+^ monocytes expressing high levels of myeloid markers such as *FCGR3A*, and monocyte DCs cells characterised by high *CD1C*, *FCER1A,* and *CLEC10A* expression (Fig. 5b). As shown in Fig. 4c, the proportion of CD14^+^ monocytes in AML patients (AML2, and AML5) was significantly higher than that in healthy controls. The results showed that patients with AML exhibited a significant increase the number of CD14^+^ monocytes, indicating an increase in pro-inflammatory responses. Consistent with the clinical status that AML2 and AML5 were both in myelosuppression stage after chemotherapy. This is also coordinate to the characteristics of CD14^+^ monocytes in other disease processes and suggest their potential clinical significance in assessing the AML process [44]. In contrast, there was no significant difference in the number of CD16^+^ monocytes between patients with AML and healthy controls. As for DCs, the number of DCs significantly increased in AML1, indicating an abnormally elevated immune response, consistent with the clinical diagnosis of a high-risk status (Fig. 5c).

Next, pySCENIC analysis observed upregulation of six transcription factors (*BATF, ZNF250*, *STAT5B*, *NFIL3*, *DDIT3*, and *ETS2*) associated with apoptosis and inflammation in AML2 patient, who is a high-risk patient in myelosuppression stage after chemotherapy (Fig. 5e). *BATF* was reported to have a higher expression level in the high-risk group than in the low-risk group of AML [46]. *BATF* regulates gene expression by acting as a pioneer transcription factor in lymphocytes and supports differentiation and cytokine production by Th2, Th17, follicular T helper, and Th9 cells [47, 48]. We also observed the enrichment of specific gene sets involved in the inflammatory response, apoptosis, IL6- JAK-STAT3 signalling, and IL2-STAT5 signalling in monocytes and DCs (Fig. 5d).

To explore the clonal structure of CD14^+^ monocytes and monocyte-DCs, we applied the inferCNV algorithm to analyse the CNVs of single cells. As a result, we observed a significantly higher CNV and chromosomal instability index among AML samples compared to healthy controls (Supplementary Fig. 9). For example, compared with healthy samples, the monocyte-DCs of AML1 had an obvious change, in which the CNVs of chromosome pairs 3, 5, 7, and 9 were significantly reduced, whereas the CNVs of chromosome pairs 2, 4, 11, and 19 increased, which may be caused by the increase in malignant tumor cells. Based on the aggregated CNV results of CD14^+^ monocytes, the common chromosome copies number alterations, such as the gains in chromosome 1 and the loss of chromosomes 5, 6, and 9, might be associated with chemotherapy response [49].

These findings emphasise the impact of AML on monocytes and DCs and suggest that the number of CD14^+^ monocytes, DCs, and CNVs can be used in tandem to assess AML clinical status during chemotherapy.

### Ligand–receptor analysis reveals AML interactomes in HSC-Prog

**Fig. 6:**
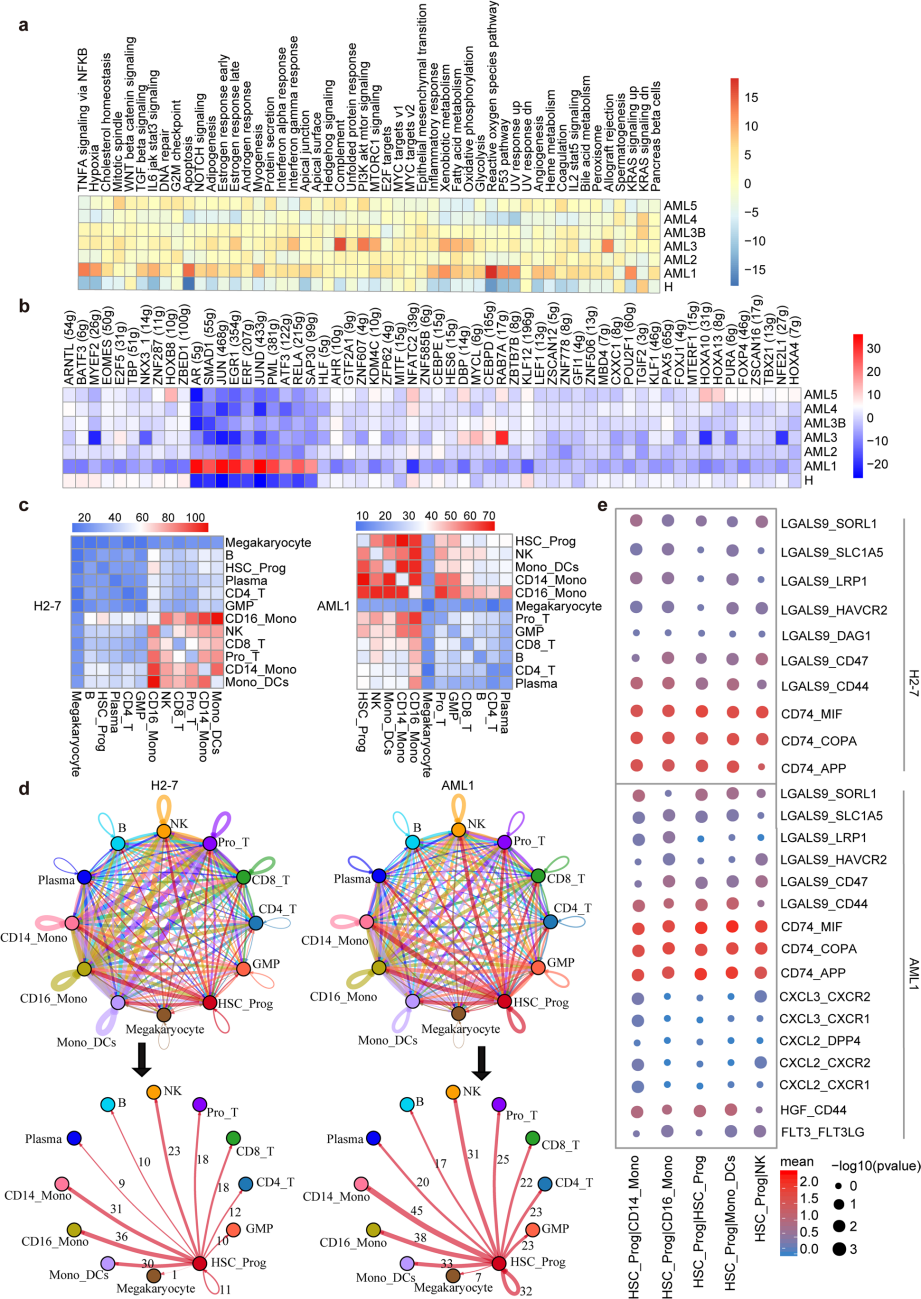
Immune landscape of HSC-Prog cells in AML and healthy samples. **a** Heatmap of 50 hallmark gene sets in the MSigDB database among the healthy and AML. **b** Heatmap of AUCell tvalue of transcription factor in HSC-Prog cells among AML patients and healthy donors. Red and blue represent upregulated and downregulated TFs, respectively. **c** Heatmap showing the total number of interactions between cell types in the decidua dataset obtained from CellPhoneDB. Red and blue represent a high and low number of interactions, respectively. **d** Connectome web analysis of lean interacting tissue-resident cell types based on the expression in the cell population. The thickness of the connecting lines is proportional to the number of interactions between two nodes. **e** Dot plot depicting selected cell–cell interactions between HSC-Prog and other clusters. The size of the dots represents the statistical significance of the interaction. Red and blue represent high and low marker expression levels, respectively

Standard chemotherapy for AML targets proliferating cells and effectively induces complete remission; however, many patients eventually experience relapse [50], which is caused by leukaemia stem cells that can self-renew. The self-renewal and proliferation of normal HSCs produce pluripotent progenitor cells (stem cells) and daughter cells that differentiate into effector cells [51]. To investigate the heterogeneity of HSCs and progenitor (Prog) cells in AML after chemotherapy, we performed GSVA analysis to compare the HSC-Prog genome in AML and healthy donors. Different from other AML samples, AML1 (a high risk AML patient in immune-recovery state after chemotherapy) upregulated in ten transcription factors, including *EGR1* (a zinc finger protein that is associated with AML progression [52]), and leukaemia transcriptional regulators *SMAD1* and *ERF* (Fig. 6b). We also observed the positive regulation of pro-inflammatory signalling pathways, including the p53 pathway, apoptosis pathway, and KRAS signalling (Fig. 6a). Upregulation of the p53 pathway may affect HSCs and transform HSCs into pre-leukemic stem cells with a significant risk of developing blood cancer, which would be concordant with the clinical results that AML1 has p53 mutations (Supplementary Table 1). AML with p53 abnormality is characterised by complex chromosomal aberrations [53]. Therefore, we performed CNV analysis on HSC-Prog using expression profiles and genomic information and noted that AML1 had an obvious gene copy number variations in most of the cell types (Supplementary Fig. 9), indicating that AML1 may produce more malignant cells.

Next, to determine the differences in structural cellular immunisations and immune–immune interactions between the cells derived from AML patients and healthy donors, we applied CellPhoneDB [54] to construct a cell–cell communication network via known ligand–receptor pairs within the 12 identified main clusters (Fig. 6c). Ligand–receptor pairs (*p* < 0.01) represent significant interactions between the two cell types. The results showed that in healthy donors, most intercellular interactions occurred between the following cell types: CD16-mono, NK, CD8^+^ T, Pro-T, CD14-mono, and mono-DCs (Fig. 6c). In contrast, in AML patients, the interaction between NK, CD14-mono, mono-DCs, and other cells was enhanced, and the range of interacting cells was expanded (Supplementary Fig. 10). Furthermore, HSC-prog cells showed more interactions with other cells through known ligand–receptor pairs, such as leukaemia-associated ligands *CXCL2*, *CXCL3*, and *FLT3*, signalling to the receptors *CXCR1* and *CXCR2* expressed by CD14-mono and NK cells [55, 56] (Fig. 6e). We also noted the enrichment of activation of the HGF-CD44 pathway between HSC-Prog and CD14-mono, CD16-mono, and mono-DCs in AML1. This suggests the role of HSC-Prog as a central communication hub in AML patients (Fig. 6d), highlighting its important role in AML development; moreover, it indicates that cell–cell communication is strengthened in the AML patient.

These findings emphasise the intra-tumoral heterogeneity at the transcriptional and cell–cell communication levels in HSC-prog, as well as highlight the need for more precise biomarkers to enhance chemotherapy efficacy. Our results also demonstrate the potential of targeting ligand–receptor pairs in HSC-Prog, such as *CXCL2* and *CXCL3* and their receptors *CXCR1* and *CXCR2*, for AML diagnosis or treatment.

## Discussion

With the advent of single-cell technology, it is now possible to examine cellular heterogeneity using transcriptomic studies. Technological advancements have expanded our understanding of the molecular mechanisms underlying benign and malignant haematopoiesis. Post-treating immune monitoring is essential for AML diagnosis and prognosis. We collected data from many cells using high-throughput scRNA-seq technology and performed an integrated post-treating immune monitoring for AML patients.

The clinical benefits of immune checkpoint blockade therapies are observable in tumors with high immune cell infiltration, particularly in haematological malignancies, such as AML. Importantly, even patients who initially respond to immune therapy cannot achieve long-term disease control, as primary and acquired resistance mechanisms are differentially orchestrated in haematological malignancies, depending on tumor type and/or genotype. Single-cell RNA sequence analysis helps identify tumor microenvironment information in detail and implements more effective immunotherapy combined with conventional chemotherapy [6, 38]. Among these, NK cell immune checkpoint molecules are potential targets for cancer immunotherapy [38]. We found that the interaction between HLA-F, HLA-E, HLA-C, and B/CD8 + T/HSC-Prog/plasma in NK cells is enhanced in AML, suggesting that these immune checkpoints may be potential targets for the future treatment of AML.

Immunotherapeutic approaches, such as stem cell transplantation and anticancer mAbs, have contributed to improving outcomes in haematological malignancies [57–59]. Many studies have revealed changes in T cell-mediated immunity in AML [60]. In this study, we identified nine T cell subsets of CD4^+^ and CD8^+^ T cells and observed enhanced signalling pathways related to TNFA, NFKB, hypoxia, KRAS, MTORC1, and other hallmark gene sets in AML patients using GSVA and GSEA. Furthermore, we showed that CD4^+^ Tregs are associated with an increased risk of recurrence, which is concordant with the results of studies using other research methods [61]. Monocytes, macrophages, and DC progeny form the basis of innate and adaptive immunity and play essential roles in regulating the immune response [43, 45]. We identified three subsets of monocytes and DCs, as well as observed that the number of CD14^+^ monocytes was proportional to the inflammatory response in patients and that the proportion of this subgroup changed with disease progression [44]. Furthermore, we found that in CD14^+^ monocytes, genes related to inflammatory response, apoptosis, and inflammatory signalling were overexpressed, in addition to five transcription factors, including BATF, which was reported to be hyper-activated in AML samples [46]. Monocyte-DCs are upregulated in AML1 patients with significant CNV changes, indicating that AML disease accelerates the differentiation of monocytes into DCs. These results highlight the heterogeneity of monocytes and the signalling pathways that may lead to the progression and recurrence of AML. Our study also revealed that HSC-Prog was highly enriched in PBMC of severe AML patient (AML1), had stronger interactions with monocytes and DCs, and overexpressed genes involved in signalling pathways associated with leukaemia, suggesting that HSC-Prog plays an important role in the process of AML.

## Conclusion

In this study, we used scRNA-seq to create a single-cell AML landscape by analysing 91,772 cells from five AML patients and six healthy donors. We identified 14 main cell clusters, including NK, monocytes, and DCs, as well as nine subclusters within CD4^+^ T, CD8^+^ T, and their corresponding gene markers. Additionally, we found that the proportion of CD8^+^ T and NK cells decreased in AML; accordingly, there exists potential for a general diagnostic method to help identify effective immunotherapy strategies by targeting CD8^+^ T and NK cells. Moreover, AML progression was associated with an increase in the number of CD14^+^ monocytes and monocyte-DCs as the CNVs changed, suggesting their potential as targets in new immunotherapeutic strategies. Importantly, we revealed that HSC-Prog exhibits great heterogeneity in chromosomal structure and intercellular signalling networks by highlighting cell–cell interactions in AML. Overall, our study reveals the detailed immune landscape of AML after therapy, thereby providing a basis for the determination of therapeutic targets and facilitating the search for effective immunotherapeutic strategies for AML. Our study also has limitations, in clinical, relapsed AML patients usually resistant to chemotherapy. The long-term follow-up of the same case, even the end-point follow-up, will have more clinical diagnostic and therapeutic value. In the next stage, we will continue on long-term monitoring of the patients under therapy.

## Methods

### Sample collection

Five patients were pathologically diagnosed with AML at Shenzhen Peoples’ Hospital, 2^nd^ clinical medicine college, Jinan University (Fig. 1a). The available clinicopathological features of these patients were summarized in Supplementary Table 1, and six PMBC samples were designated as the discovery cohort. Six PBMC samples from healthy donors were collected and processed at Shenzhen Peoples’ Hospital. All patients and healthy donors had accepted the informed consent forms, and the study was approved by the Research Ethics Committee approval (LL-KY-2021271).

### PBMC cell suspensions preparation

Lymphoprep is added to the SepMate tubes (STEMCELL Technologies) by carefully pipetting it through the central hole of the SepMate insert, 10 mL fresh blood is diluted by two folds with PBS and layered on the top of Lymphoprep. The tubes were then centrifuged at 1200 × g for 10 min at room temperature, with the brake on. After the centrifugation, the plasma is at the top, followed by the PBMC/platelet layer, the Lymphoprep layer, the granulocyte layer, and finally the erythrocytes at the bottom of the centrifuge tube. The upper plasma layer was drawn into 15 mL centrifuge tube using a sterile pipette, the PBMC layer was transferred into a new 1.5 mL centrifuge tube, using another clean pipette. The PBMCs were washed by adding five volumes of PBS, centrifuged at 400 × *g* for 5 min at 4°C, and the supernatant was removed. 1.25 mL of red blood cell lysis buffer was then added to the pellet and mixed gently by slowly pipetting up and down for five times. The PBMCs were allowed to stand on ice for 8 min and then centrifuged at 300 × g for 5 min at 4°C. The supernatant was carefully removed without disturbing the pellet, followed by resuspension of pellet in 1 mL PBS by slowly pipetting up and down for five times to wash the cells. The cells were centrifuged at 300 × g for 5 min at 4°C, followed by another wash with PBS. The cells were then resuspended with PBS, and manually counted for three times by Trypan blue exclusion. Finally, the cells were centrifuged and resuspended at a concentration of 700–1200 cells/µl used for scRNA-seq.

### scRNA-seq library preparation and sequencing

Single-cell suspensions were loaded into Chromium microfluidic chips using the Chromium Single Cell 3ʹ GEM, Library & Gel Bead Kit v3 (10X Genomics) according to the manufacturer’s instructions. All the subsequent steps were performed following the standard manufacturer’s protocols. The barcoded library was sequenced on the NovaSeq6000 sequencer (Illumina) with 150 bp paired-end reads by Novogene (China).

### scRNA-seq data processing and analysis

The raw scRNA-seq data were aligned to the human reference genome (GRCh38) and a digital gene expression matrix was built using STAR algorithm in CellRanger (‘count’ option; v3.1.0; 10 × Genomics). The ‘filtered_feature_bc_matrix’ file folder generated by CellRanger was used for further analysis. Using the Seurat R package [62] (v3.1.0; https://satijalab.org/seurat), cells that contained more than 5500 or fewer than 200 expressed genes, or more than 15% mitochondrial transcripts, or more than 5% hemoglobin transcripts were removed. Genes that were expressed in fewer than 3 cells were also removed. In addition, doublets identified by DoubletFinder [63] (version 2.0.2; https://github.com/chris-mcginnis-ucsf/DoubletFinder) were also dropped. Following the removal of the poor-quality cells and doublets, a total of 91,772 cells were retained for downstream analysis.

For each cell, the expression of each gene was normalized and log-transformed using ‘NormalizeData’ function (scale.factor = 10,000) of Seurat. Then, the 12 samples were integrated by ‘FindIntegrationAnchors’ and ‘IntegrateData’ with 2000 variable genes. After data scaling using ‘ScaleData’, principal component analysis and UMAP analysis were performed for dimension reduction by ‘RunPCA’ and ‘RunUMAP’, respectively. Finally, cell clusters were identified using ‘FindNeighbors’ and ‘FindClusters’ (resolution = 3). The ‘FindAllMarkers’ function in Seurat was used to find markers of each identified cluster. Then, clusters were annotated based on the expressions of canonical markers in particular cell types [15, 64].

### Functional Enrichment Analysis

Different expression genes (DEGs) were identified using the ‘FindMarkers’ or ‘FindAllMarkers’ function in Seurat with default parameter. DEGs were filtered using a minimum log (fold change) of 0.25 and p_val_adj < 0.05. Gene set variation analysis (GSVA) was performed using 50 hallmark gene sets obtained from the molecular signature database (http://www.gsea-msigdb.org/gsea/msigdb) by the GSVA package (v1.34.0) [64].

### Cell–cell communication analysis

Cell–cell communication was analyzed using CellPhoneDB (v2.1.1, https://github.com/Teichlab/cellphonedb) with normalized count data as input file according to the literature [54]. The significant ligand-receptor pairs were filtered with a P value of less than 0.05.

### pySCENIC analysis

Regulons of transcription factors (TFs) and their target genes were analyzed by pySCENIC (v0.10.0, https://github.com/aertslab/pySCENIC) with raw count matrix as input [65]. Briefly, the coexpression modules were inferred by GRNBoost2, then the regulons were identified by cisTarget, and the activity of these regulons was quantified by AUCell.

### Copy-number variation (CNV) analysis

Single-cell CNV analyses were estimated by the inferCNV package (v1.2.0; https://github.com/broadinstitute/inferCNV/wiki) with a raw count matrix as input. The cells of the same type in the healthy group were used as reference. In addition, the parameters of inferCNV analysis were set as default.

### Supplementary Information

Below is the link to the electronic supplementary material.

Supplementary file1 (PDF 367 KB)

Supplementary file2 (PDF 6263 KB)

Supplementary file3 (PDF 7468 KB)

Supplementary file4 (PDF 2213 KB)

Supplementary file5 (PDF 7169 KB)

Supplementary file6 (PDF 6100 KB)

Supplementary file7 (PDF 2329 KB)

Supplementary file8 (PDF 2515 KB)

Supplementary file9 (PDF 10574 KB)

Supplementary file10 (PDF 2492 KB)

Supplementary file11 (XLSX 10.5 KB)

Supplementary file12 (CSV 1 KB)

Supplementary file13 (CSV 2 KB)

Supplementary file14 (CSV 1 KB)

## Data Availability

scRNA-seq data have been uploaded to the Gene Expression Omnibus repository: https://www.ncbi.nlm.nih.gov/geo/query/acc.cgi?acc=GSE235857.
