# Supplementary figures and images for: Single-cell transcriptomic profiling reveals immune cell heterogeneity in acute myeloid leukaemia peripheral blood mononuclear cells after chemotherapy
Source: Cell Oncol (Dordr). 2023 Aug 24;47(1):97–112. doi: 10.1007/s13402-023-00853-2 (PMC10899424; doi:10.1007/s13402-023-00853-2)

**a**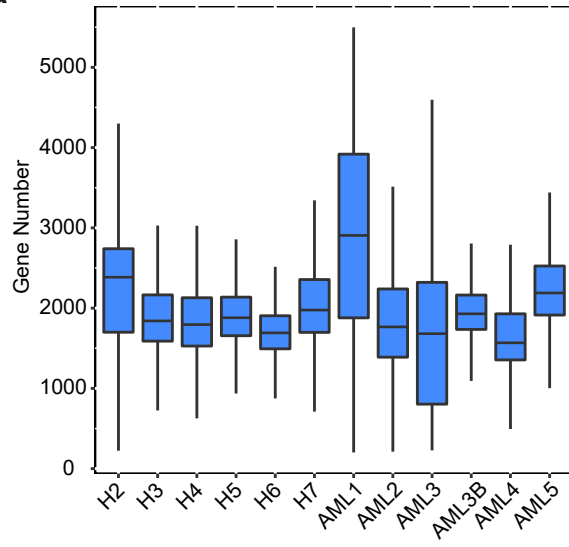**b**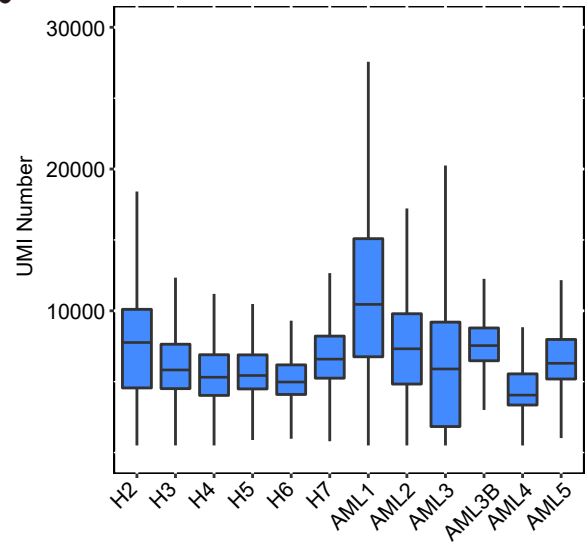

Supplement: Supplementary file 1 — Supplementary file1 (PDF 367 KB) [file 13402_2023_853_MOESM1_ESM.pdf]

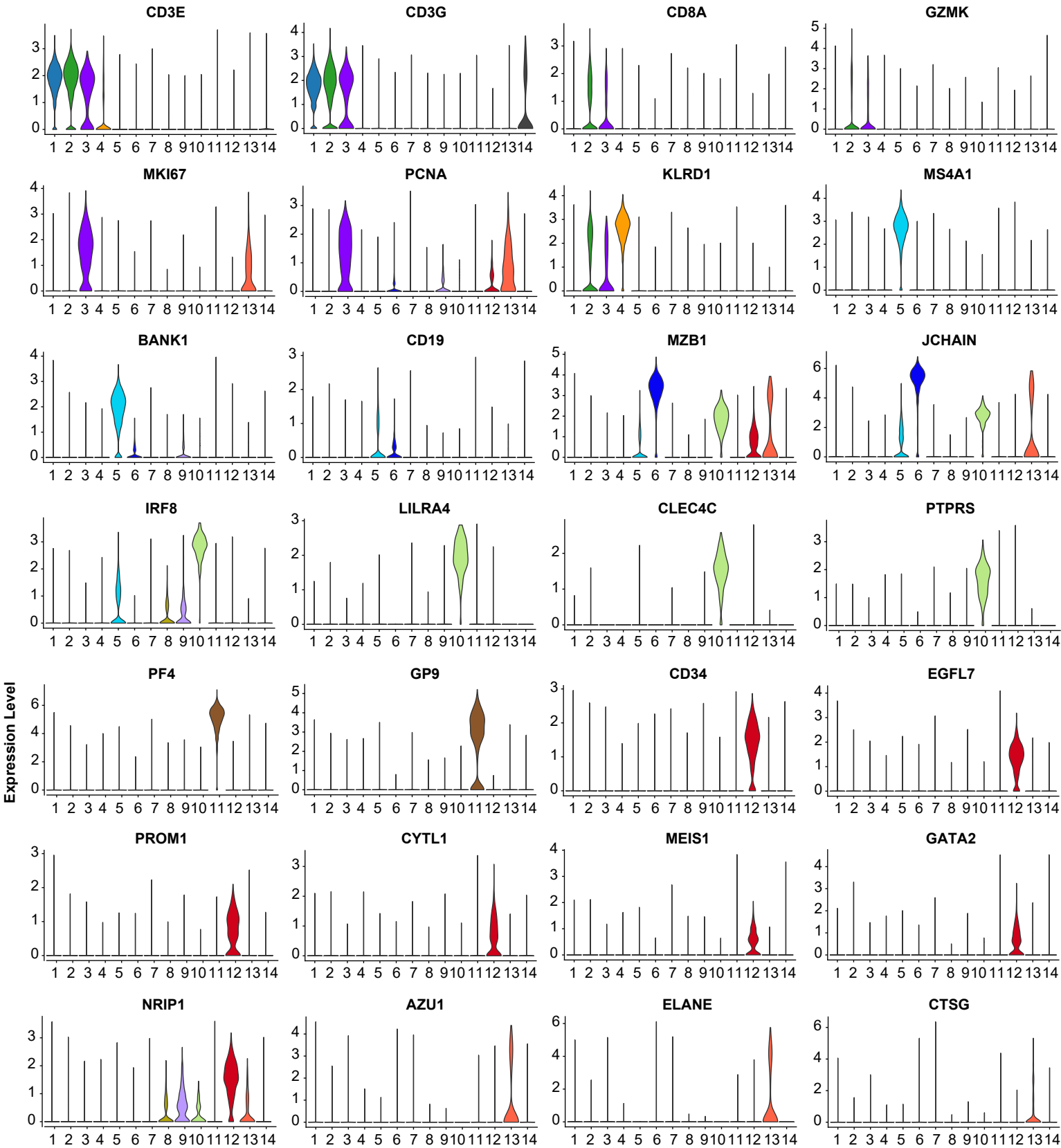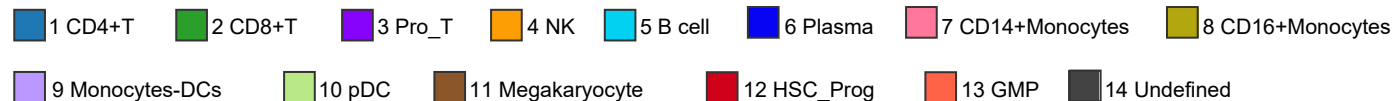

Supplement: Supplementary file 2 — Supplementary file2 (PDF 6263 KB) [file 13402_2023_853_MOESM2_ESM.pdf]

**a**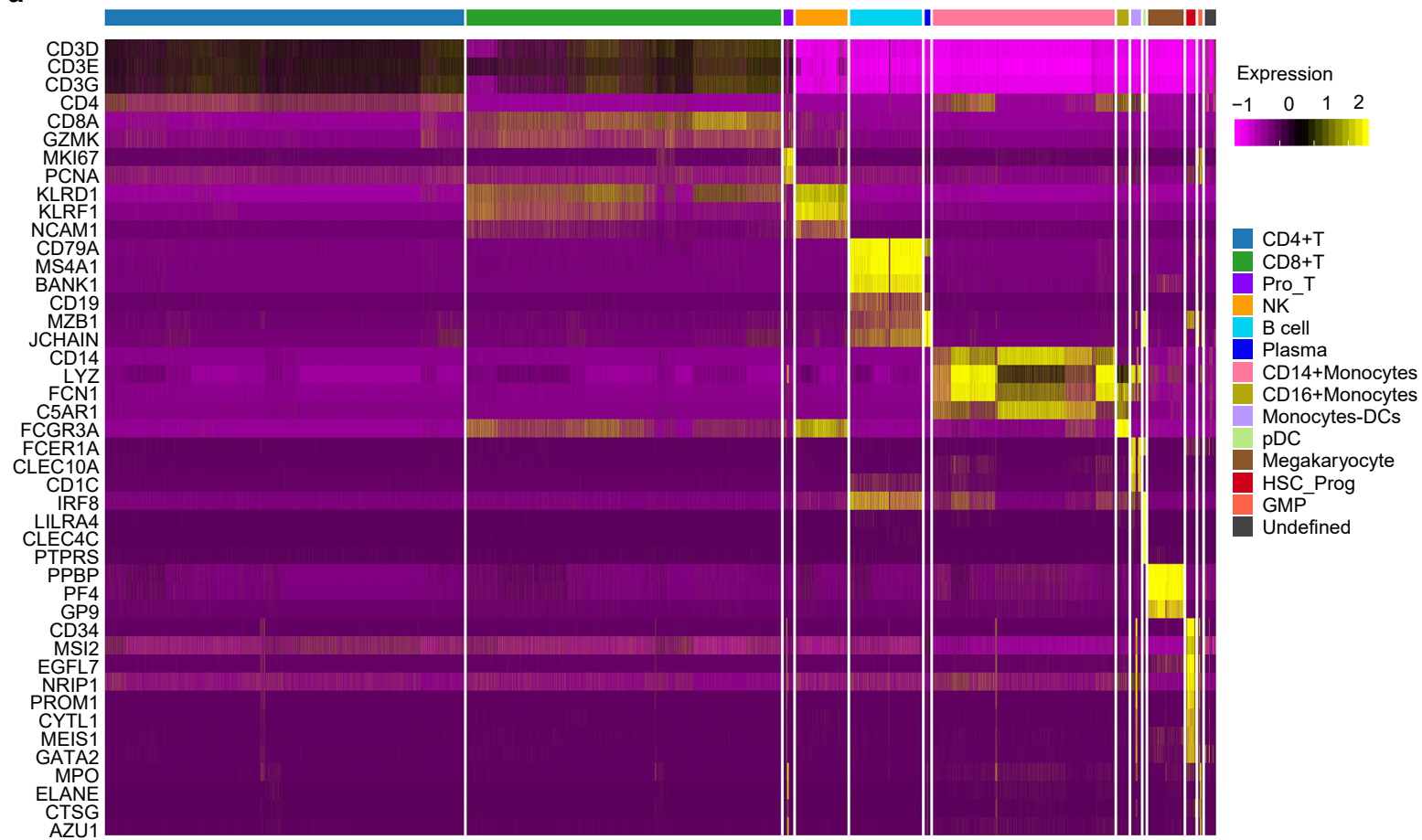**b**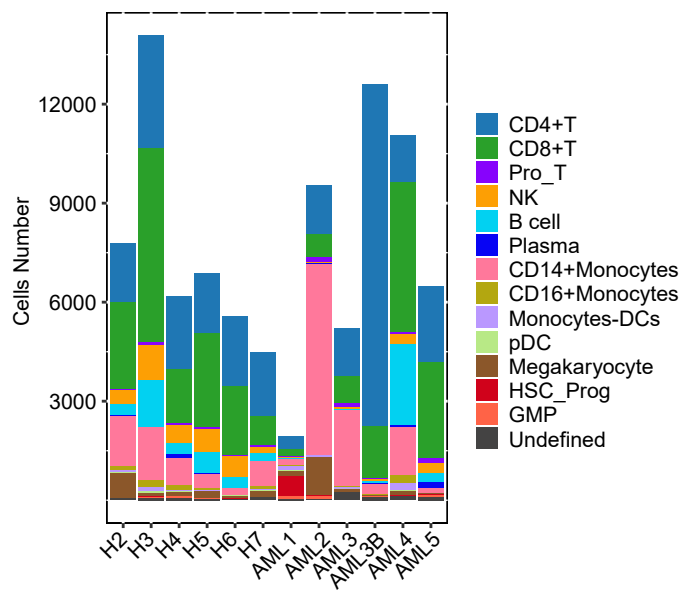

Supplement: Supplementary file 3 — Supplementary file3 (PDF 7468 KB) [file 13402_2023_853_MOESM3_ESM.pdf]

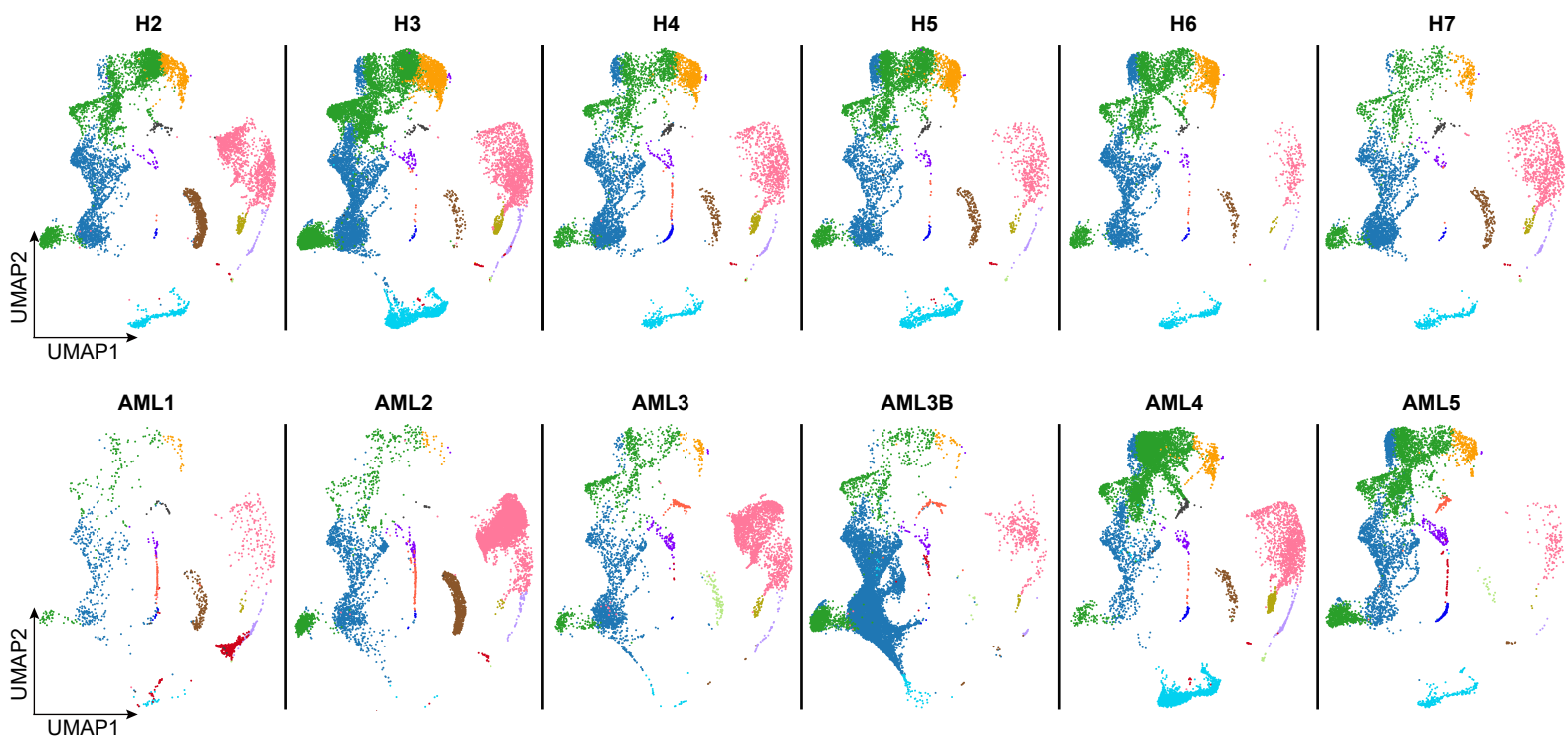

Supplement: Supplementary file 4 — Supplementary file4 (PDF 2213 KB) [file 13402_2023_853_MOESM4_ESM.pdf]

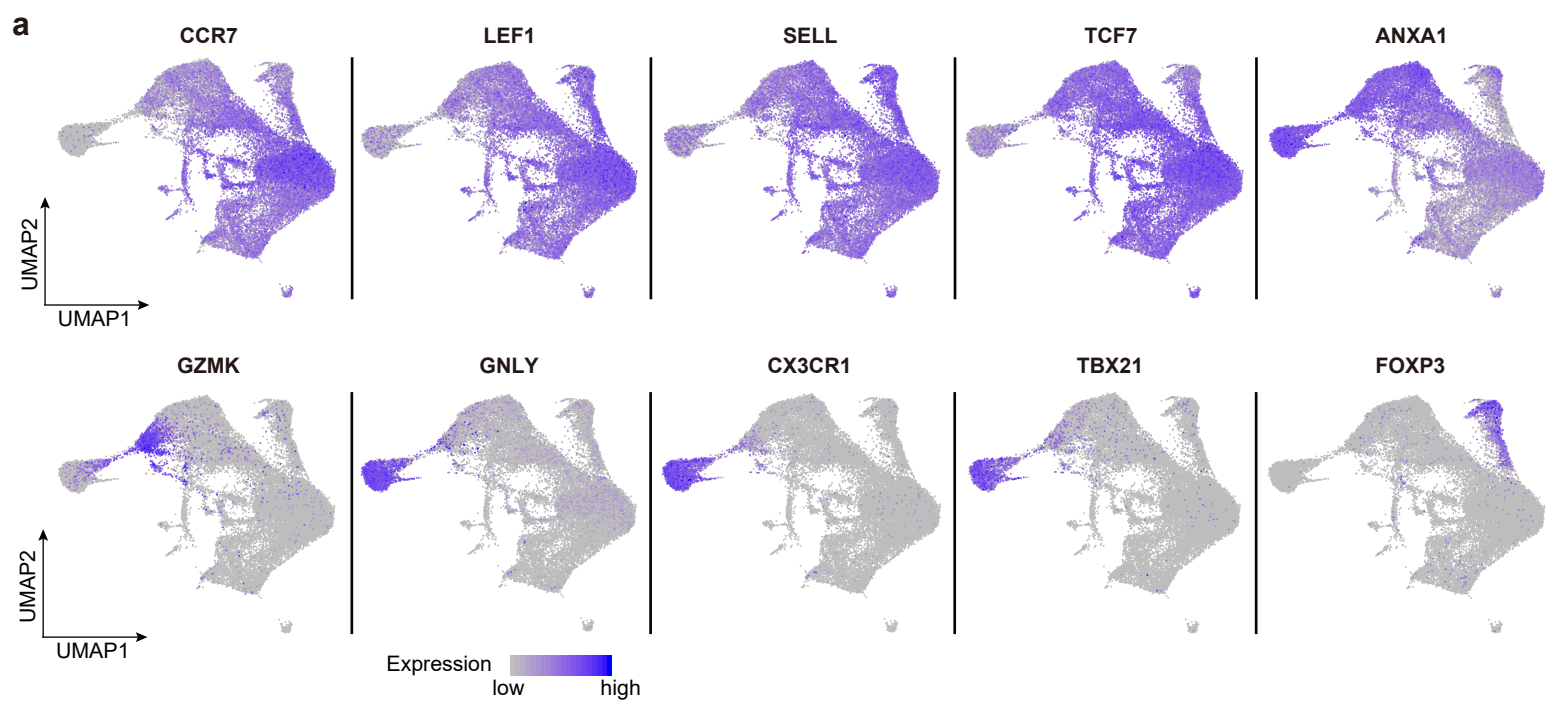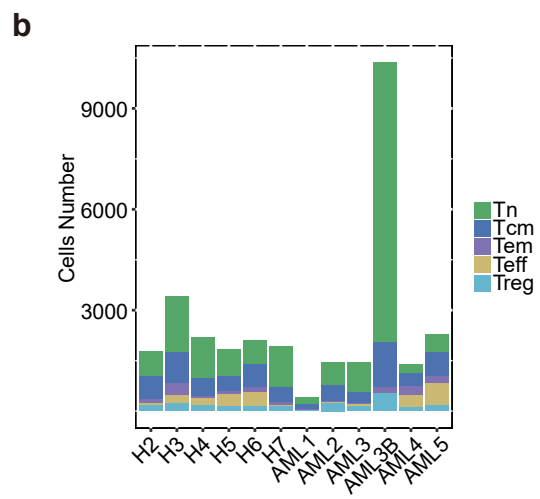

Supplement: Supplementary file 5 — Supplementary file5 (PDF 7169 KB) [file 13402_2023_853_MOESM5_ESM.pdf]

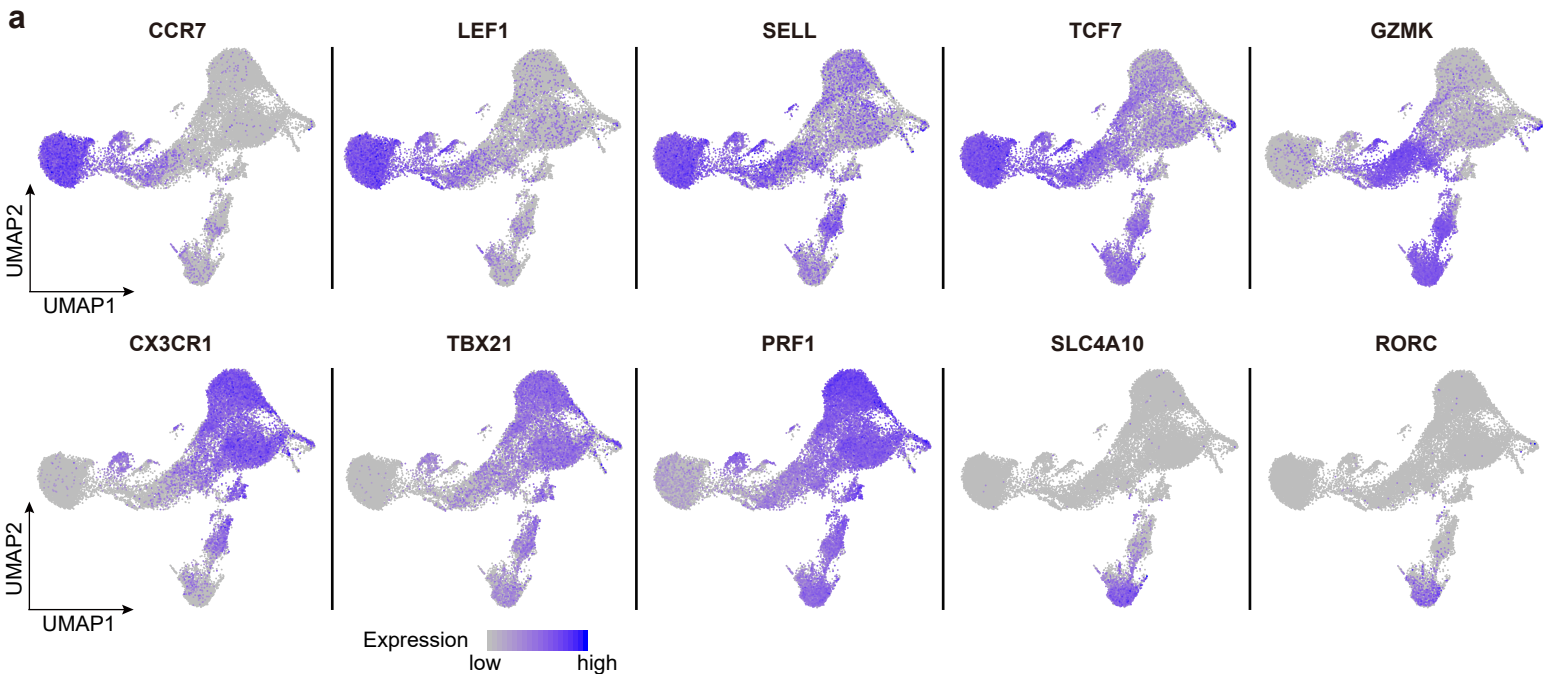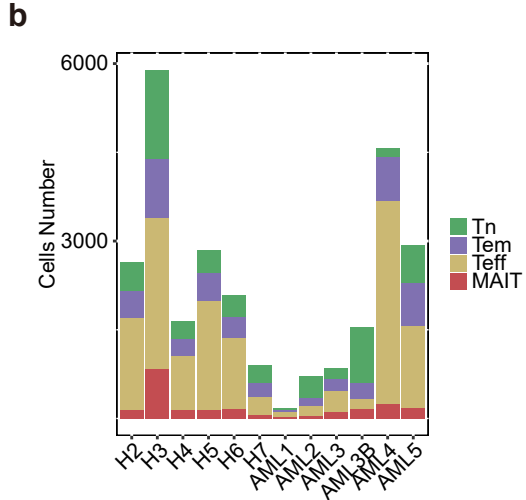

Supplement: Supplementary file 6 — Supplementary file6 (PDF 6100 KB) [file 13402_2023_853_MOESM6_ESM.pdf]

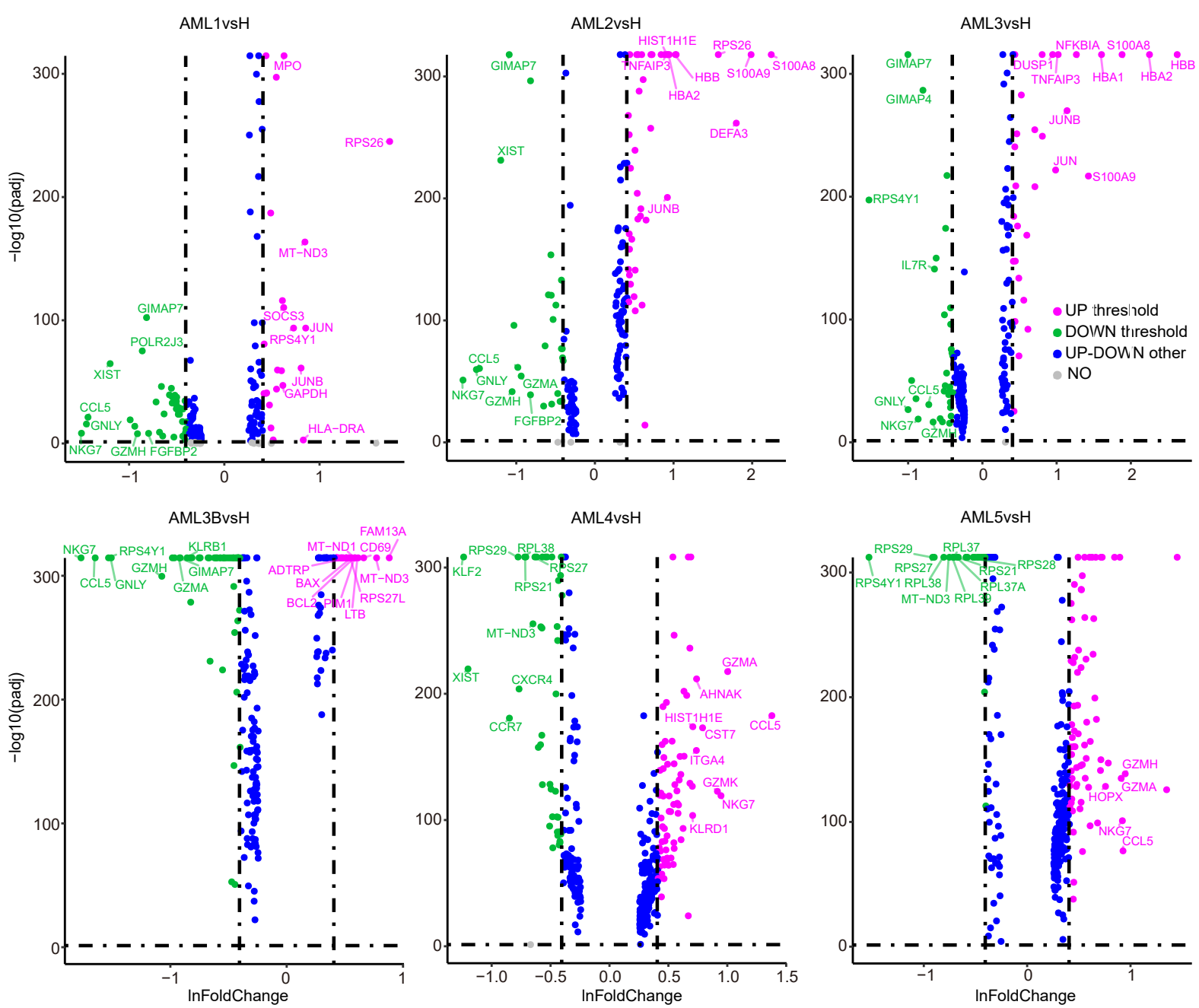

Supplement: Supplementary file 7 — Supplementary file7 (PDF 2329 KB) [file 13402_2023_853_MOESM7_ESM.pdf]

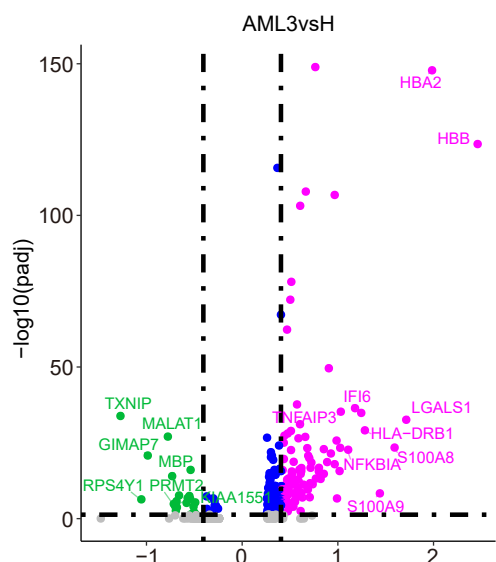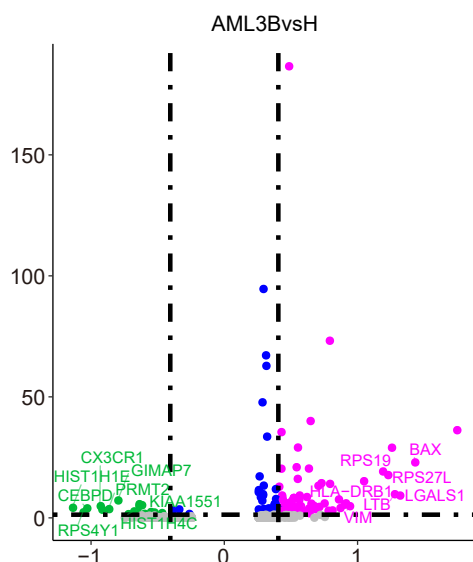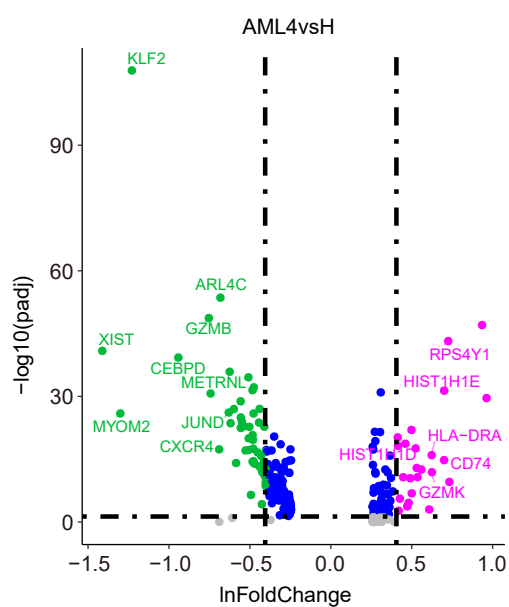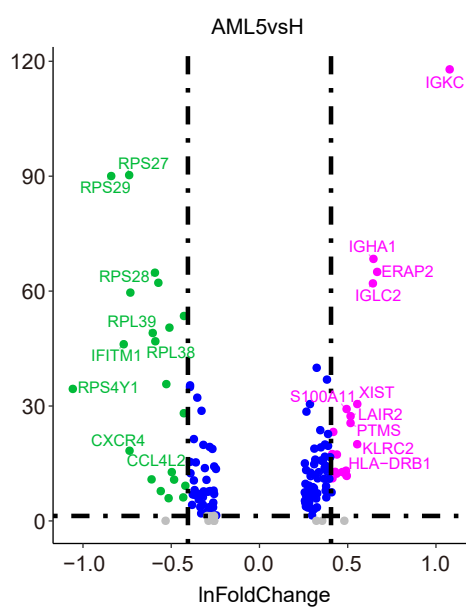

Supplement: Supplementary file 8 — Supplementary file8 (PDF 2515 KB) [file 13402_2023_853_MOESM8_ESM.pdf]

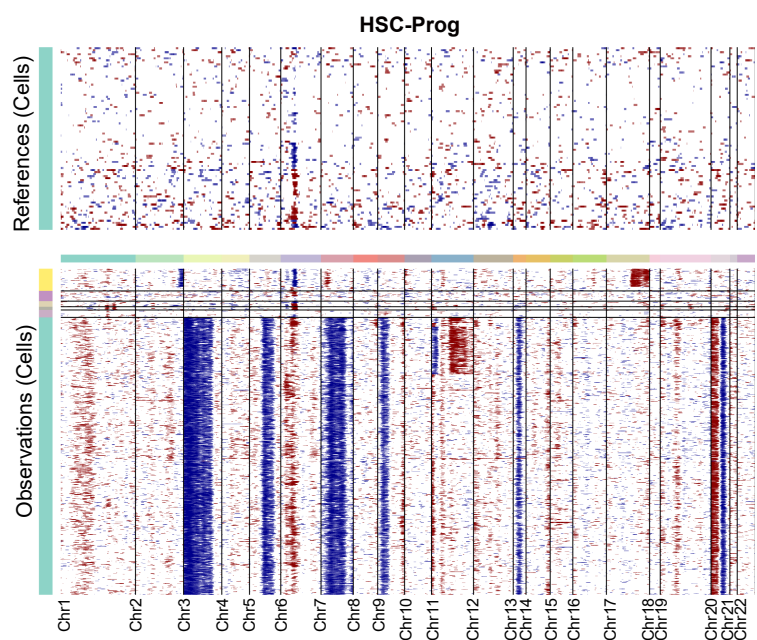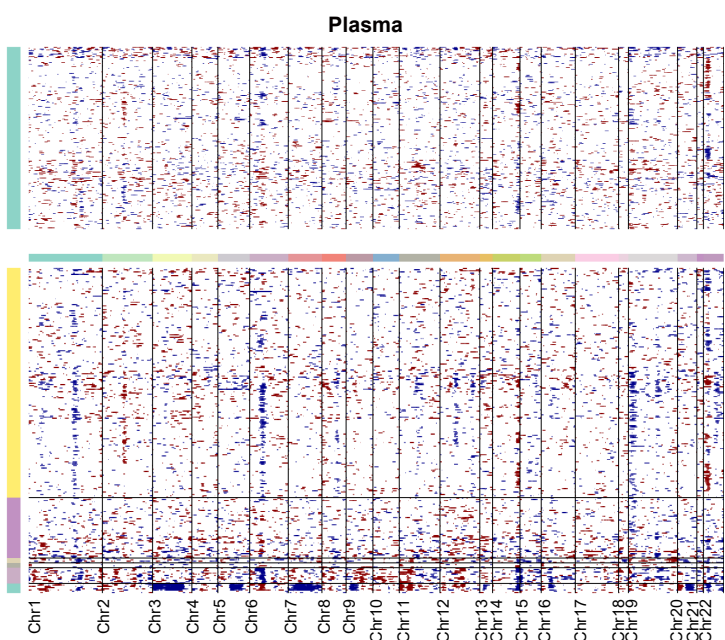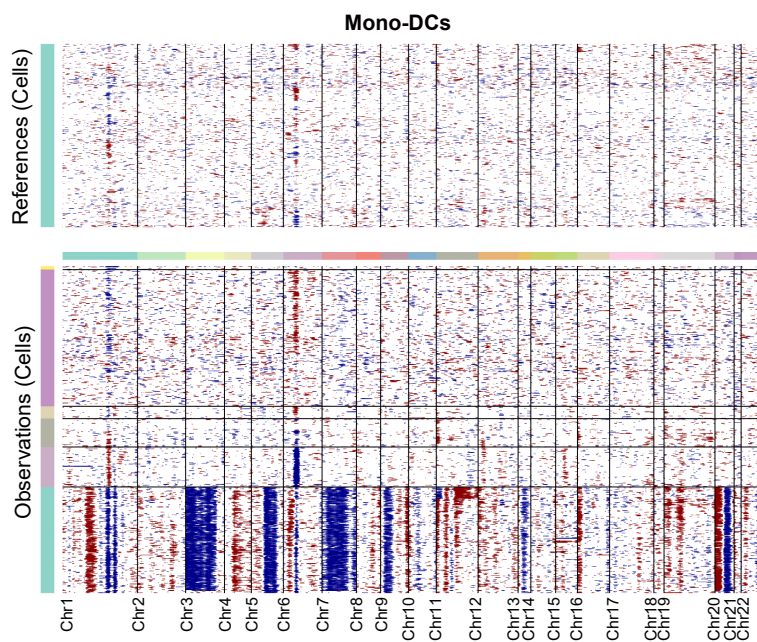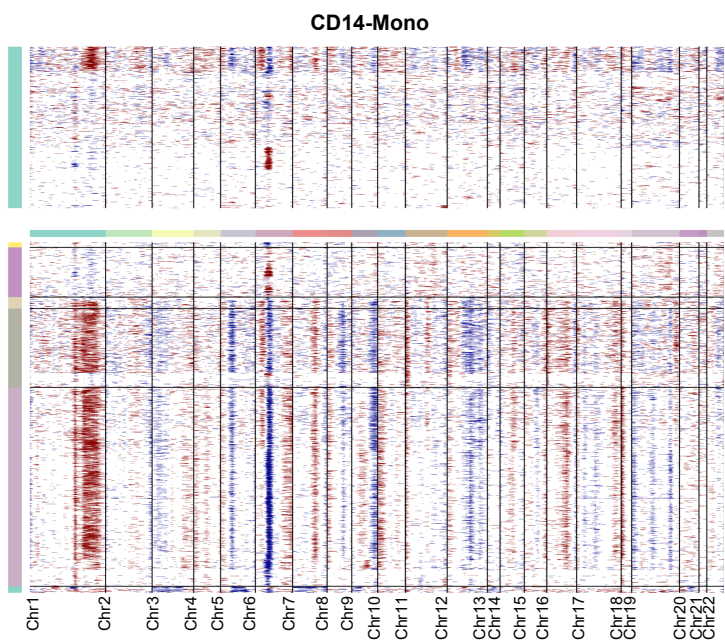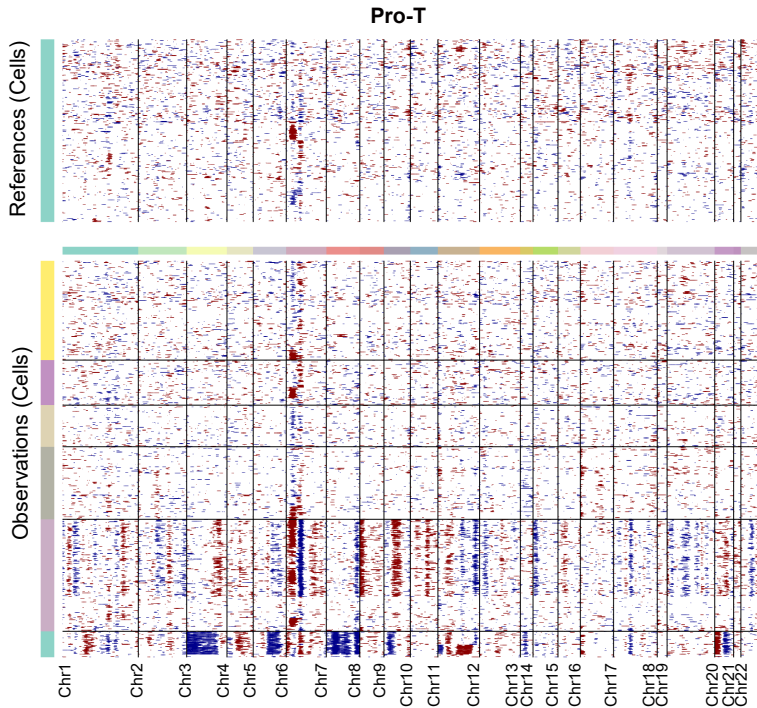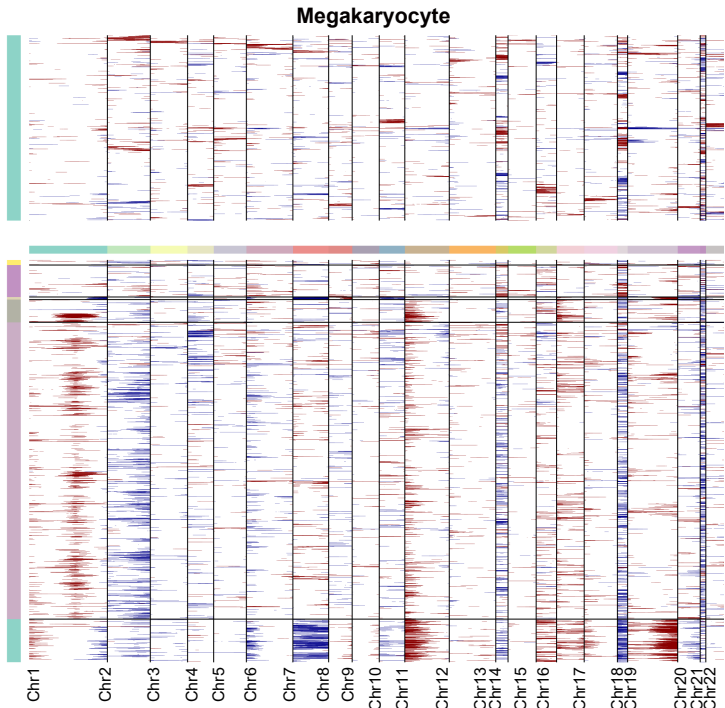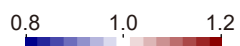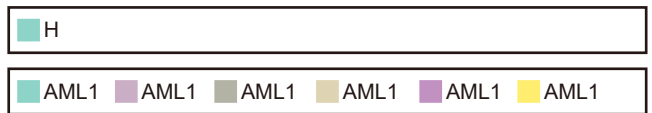

Supplement: Supplementary file 9 — Supplementary file9 (PDF 10574 KB) [file 13402_2023_853_MOESM9_ESM.pdf]

**a****AML2**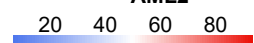**AML3**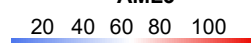**AML3B**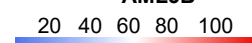**AML4**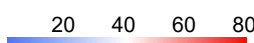**AML5**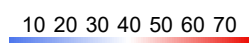**b****AML2**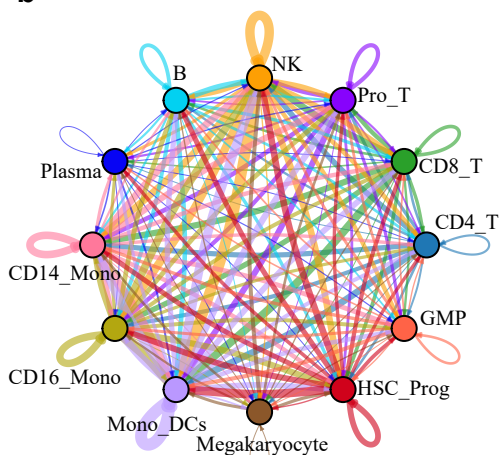**AML3**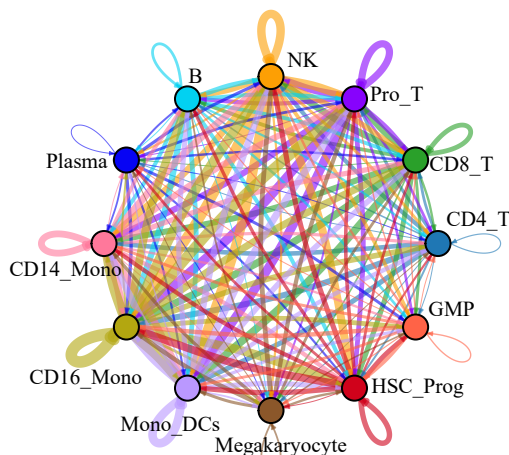**AML3B**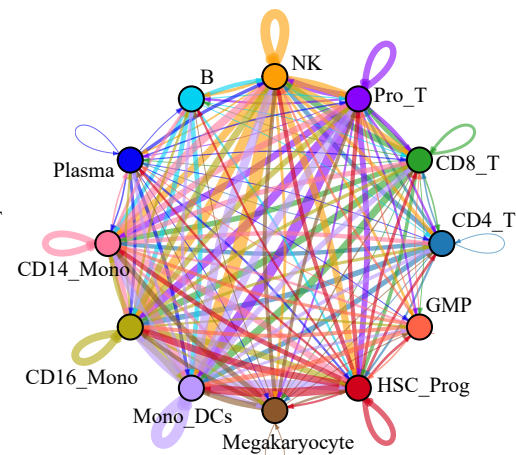**AML4**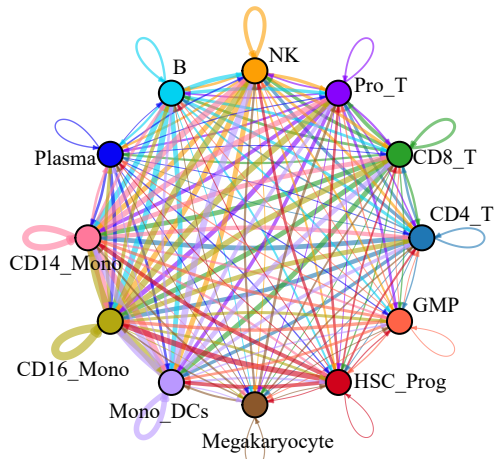**AML5**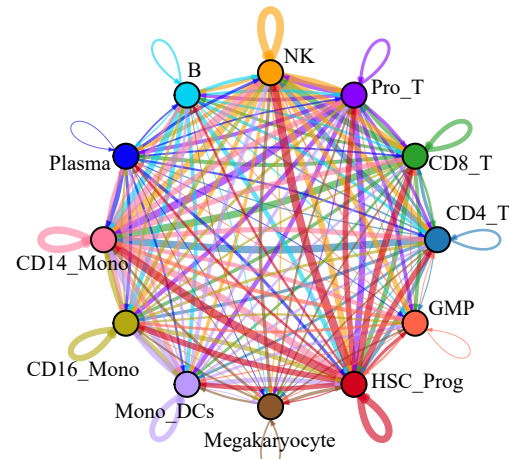

Supplement: Supplementary file 10 — Supplementary file10 (PDF 2492 KB) [file 13402_2023_853_MOESM10_ESM.pdf]
